# Remote Reflectivity Sensor for Industrial Applications

**DOI:** 10.3390/s21041301

**Published:** 2021-02-11

**Authors:** Federico Cavedo, Parisa Esmaili, Michele Norgia

**Affiliations:** Department of Electronics, Information and Bioengineering Politecnico di Milano, 20133 Milan, Italy; federico.cavedo@gmail.com (F.C.); parisa.esmaili@polimi.it (P.E.)

**Keywords:** reflectivity measurement, laser sensors, remote encoder, object recognition, distance measurement, digital baseline restorer

## Abstract

A low-cost optical reflectivity sensor is proposed in this paper, able to detect the presence of objects or surface optical properties variations, at a distance of up to 20 m. A collimated laser beam is pulsed at 10 kHz, and a synchronous digital detector coherently measures the back-diffused light collected through a 1-inch biconvex lens. The sensor is a cost-effective solution for punctual measurement of the surface reflection at different distances. To enhance the interference immunity, an algorithm based on a double-side digital baseline restorer is proposed and implemented to accurately detect the amplitude of the reflected light. As results show, the sensor is robust against ambient light and shows a strong sensitivity on a wide reflection range. The capability of the proposed sensor was evaluated experimentally for object detection and recognition, in addition to dedicated measurement systems, like remote encoders or keyphasors, realized far from the object to be measured.

## 1. Introduction

Contactless optical measurements are gaining significant attention for feature extraction of an object, and also for the growing needs of various industrial applications, such as computer-numerical control (CNC) machines [1,2], robot control [3], automated driving applications [4], and tracking systems [5,6]. Considering in-line measurement, non-invasive instruments are dominant due to their advantages over invasive techniques, including flexible design, reduced installation complexity, and relatively short measurement times without the risk of damage specifically in surface analysis [7]. As a promising solution in position measurement, specifically displacement measurement, heterodyne phase-detection based laser interferometry instruments have been widely used in industries [8,9], even if their cost can be very high. Despite their long displacement range with sub-nanometer resolution, the performance is degraded due to high sensitivity to environmental conditions and consequently they have failed to mitigate in-line measurement specification [10]. A different approach is given by optical triangulation [11,12,13], and this may be the best solution for short distances, but it is difficult to implement for long distances with high accuracy, because it essentially consists of a measure of the angle of view. Another limitation concerns the optical accessibility, as a triangulation system needs an outreach between the transmitter and the receiver, which prevents the measurement in holes or partially obscured areas. As a highly robust technology, digital image correlation (DIC) has been widely accepted and used in displacement and deformation measurements [14,15]. In such approaches, the displacement can be determined by analyzing the correlation of digital images for the target taken before and after displacement [16]. In the case of large displacements, a line-scan camera with a large image sensor is selected instead of a conventional 2D (two-dimensional) area-scan camera as the displacement reader, although, a subpixel image registration method needs to be implemented to improve the accuracy. Peak location search in the cross-correlation of the two aligned images is widely used for subpixel image registration [17]. Concerning the computational speed and memory requirements, the Fourier domain approach is preferred. A single-step discrete Fourier transform (SSDFT) is proposed that uses the matrix-multiplied discrete Fourier transform (DFT) algorithm to compute the up-sampled cross-correlation between the reference and sensed images [18]. In [19], a position measurement system is proposed based on the 1D SSDFT subpixel image registration algorithm. The system consists of a patterned target attached to a linear stage and line-scan camera as a displacement reader. The system shows ± 0.3 µm within 50 mm. Despite the cost and level of complexity in such vision-based distance and displacement measurement techniques, the short measurement range limits their application where the target is placed relatively far. Due to their simplicity and therefore low cost, intensity-based sensors are widely used in optical distance measurement systems. The system consists of a light source and a detector, in which the light intensity reflected from the object onto the detector is a function of absolute reflectance and the diffusivity of the object in addition to the distance between the light source/detector and the object. This is the simple principle behind the reflective-type optical encoders [20] which are preferably used as reliable devices for precise displacement measurement in a variety of applications, due to their excellent immunity to electromagnetic interference, high resolution, and compact design [21]. Other remote reflectivity sensors are based on optical fibers; typically these are developed for very-short distances [22] or for selective detection of chemical species [23,24]. A summary of order of magnitude for measurement performances of different optical technologies is reported in Table 1. The last column indicates if the sensor requires a viewing angle or can measure from a single point-of-view. This feature can be important for some applications, like for example, measuring the depth of a hole.

In this paper, an instrument for reflection measurement is proposed, with the aim of obtaining a high distance range, minimum costs, and strong immunity to electromagnetic disturbances and ambient light. It is able to detect the presence of objects or surface color variation, at a distance of up to 20 m, in addition to the displacement measurements if applied as a remote encoder. The proposed instrument consists of a semiconductor laser driven by a microcontroller that sends out red light pulses. The pulses are collimated by a transmitter lens. Part of the light signal which is reflected by the target is collected through a receiver lens, on a photodiode where it is transduced in electric signal. To optimize the amount of collected light, the diameter of the receiver lens plays a significant role since the amount of received light by the photodiode is proportional to the square of its ray. In this work, a biconvex lens of 1 inch and focal length 1 inch is used to comply with requirements. The same considerations about size are also true for photodiode choice where a wide sensitive area provides good tolerance in the instrument assembly phase. To achieve enhanced interference immunity performance, an algorithm based on a double-side digital baseline restorer is proposed and implemented. Its higher performances are demonstrated in comparison with a single-side baseline restorer. As a result of implementing the proposed algorithm, the developed sensor shows a robust performance against ambient light and electromagnetic disturbances. To investigate the capability of the proposed instrument for different applications, the sensor is characterized for object detection and recognition at long distances in addition to a remote encoder—a chopper disk and spinning gray tone gradient target. As a final application, with a known target, it is possible to estimate its absolute distances, measuring the optical back-reflection.

## 2. System Description

**Figure 1:**
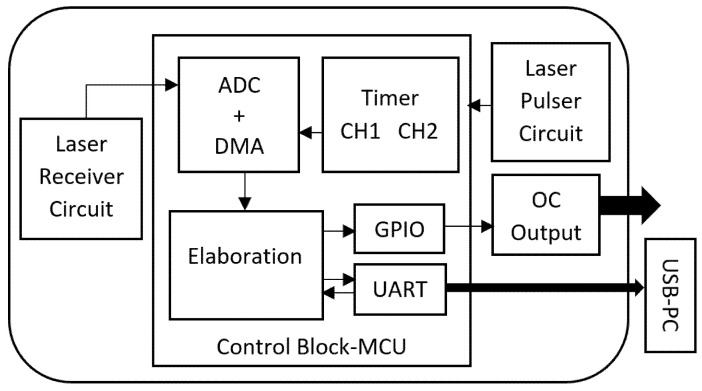
Block diagram of the proposed reflective-type displacement sensor.

**Figure 2:**
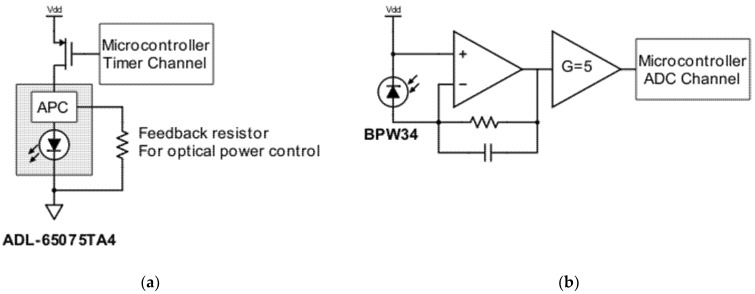
(**a**) Laser pulse generation circuit diagram including the APC unit; and (**b**) circuit diagram of the receiver.

The block diagram of the proposed reflective-type displacement instrument is represented in Figure 1. As shown in this figure, it consists of three main blocks—laser pulse generation, optical receiver, and control blocks. Since the measurement is based on evaluating the amount of reflected light from the target surface, which is directly proportional to the optical power of transmitted signal, it should be as stable as possible. Taking a close look at the laser pulse generation block, represented in Figure 2, a 650 nm laser diode (model ADL-65075TA4) is used as source of light which is driven through a high side switch by the microcontroller. Pulse energy, repetition rate, and duty cycle are selected as a trade-off between laser semiconductor life, safety class requirements, and performances in terms of resolution and output updating frequency. To achieve high sensitivity and be able to measure on a wide range, a solution with high power laser light is mandatory.

However, safety Class 2 allows optical power in the visible spectrum only up to 1 mW. This issue can be overcome by pulsing the laser diode with a small duty cycle to keep the average optical power below 1 mW and achieve a pulse with enough energy for good signal-to-noise ratio. By pulsing the laser with a pulse repetition rate of 10 kHz and a duty cycle of 10%, not only the laser safety Class 2 is guaranteed but it is also possible to achieve good sensitivity up to 20 m. To achieve robust and reliable performance, a laser diode with integrated automatic power control (APC) is required. The APC is configured to provide 10 mW optical power pulse by connecting a 3 kΩ as feedback resistor (Figure 2a). The receiver block, shown in Figure 2b, is composed of a silicon PIN photodiode (BPW34, squared with side 3 mm), a transimpedance amplifier with 270 kΩ as feedback resistor, and a second stage that provides additional factor-five gain. The light is collected through a 1-inch biconvex lens, to achieve desired sensitivity at different distances and surfaces of target. The control block is implemented by a microcontroller (STM32F303), an ARM Cortex-M4 core which is running at 72 MHz with good mixed-signal capability. Its main tasks are divided into laser pulse generation, acquisition of signal from the analog front-end, and management of the synchronization between optical pulses, sampling, and signal elaboration. Finally, the output of the proposed sensor is provided through a USB interface and an open collector (OC) output for direct industrial applications, such as keyphasors, encoders, piece counters, or intrusion detectors. The OC output can be directly connected to already-existing systems that it has been tested in

**Figure 3:**
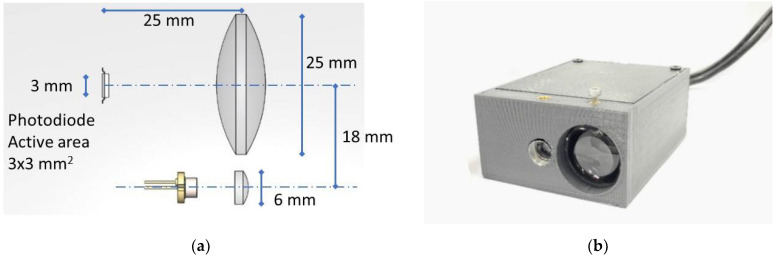
(**a**) The optical setup is composed of laser diode, transmitter lens, photodiode, and receiver lens. the (**b**) Photo of pulse amplitude estimation algorithm based on the double-side digital baseline restorer.

In terms of pulse repetition rate ($f_{r}$ = 10 kHz) and duty cycle (*D* = 10%), timing of the laser is managed by a channel of a timer in the microcontroller. The associated pin of the selected channel drives a PNP transistor which acts as a high side switch on the supply of the laser diode. The optical signal detected by the analog front-end is acquired by the internal analog-to-digital converter (ADC) of the microcontroller. It should be noted that the acquisition should be synchronized with generated laser pulse to measure the amplitude of the reflected pulse. To achieve this, a second channel of the selected timer is used to trigger the ADC. A different duty-cycle with respect to the one used to pulse the laser allows the received signal to be acquired even in the moments before the pulse. This results in a better estimation of peak value of the amplitude which will be discussed later in the pulse amplitude detection algorithm. At every trigger event, synchronous with laser pulse, a sequence of 200 samples with a resolution of 12 bits at rate of 3.5 MSPS (mega sample per second) is acquired. A direct memory access peripheral (DMA) is used to transfer every acquired sample from the ADC data buffer to the memory without the intervention of the microcontroller core. To process every generated laser pulse while achieving an output update rate of 10 kHz, a double buffer architecture is implemented. This allows the DMA to save the acquired signal related to the current pulse while the core elaborates the previous one without losing any pulse information. During the elaboration phase, the microcontroller’s task is to determine the amount of light reflected from the target, i.e., to estimate the amplitude of the pulse at output of the analog front-end. Once the amplitude of received pulse is established, the value is compared to user-defined settings to drive the open collector output. If the sensor is connected to a personal computer (PC), the microcontroller should complete other two tasks. The first task is to send the elaboration results, in addition to the sensor status, to the PC through a USB connection. The second task is to update the configuration of the sensor and save it on its own flash memory in the case of new settings received from the PC. The main setting is the threshold value for the digital open-collector output. The USB communication between the sensor and the PC is accomplished by the DMA peripheral to avoid wasting core resources that can be available for elaboration tasks. Figure 3a shows the optical setup used in this paper while the 60 × 60 × 35 mm prototype of the proposed sensor is illustrated in Figure 3b. Considering the size of the components, the procedure for optical alignment is trivial—the photodetector and lens are placed as described in the drawing, while the laser diode collimator is adjusted in order to have a well-collimated laser spot on the required operating range. It should be noted that the nature of measurement and the desired performance arrange the selection of size components, such as the receiving lens or photodiode, as mentioned earlier. The actual geometry is designed in order to work for distances from about 30 cm to 20 m, but it could be easy to design the optics for different ranges. Obviously, to work at greater distances, it is necessary to increase the size of the receiving optics to improve the received signal, and the size of the collimator, in order to decrease the angular divergence of the laser beam.

In terms of sensing and measuring approach, the main objective is to measure the amount of light reflected by the surface of target due to the laser, which is then received on the photodiode. To achieve this, two approaches can be considered. As a first approach, it is possible to sum the acquired samples corresponding to the received pulse. However, this approach can be affected by environment light added to the pulse. Since the environment light signal contribution is same with or without the laser pulse, a digital baseline restorer is implemented as a second approach. A simple realization is done by subtracting sum of the same number of samples taken after the pulse from the previous value. Equation (1) represents the mathematical expression and finite impulse response (FIR) implementation of this algorithm—the sum of 28 points during the pulse, minus the sum of 28 points during the baseline, before the pulse start.

(1) $$H\left(z\right)\;=\;-\overset{27}{\underset{i=0}{\sum }}z^{-i}+\overset{10+28+27}{\underset{i=10+28}{\sum }}z^{-i}.$$

In order to skip the transient, 10 points are left between the baseline and the pulse. Despite its reliable performance in the presence of low-frequency environment light fluctuation, the performance gets worse if a typical 100 Hz sinusoidal interference is superimposed to the pulse signal. In this paper, an algorithm is proposed and implemented to achieve enhanced interference immunity performance. In the proposed algorithm, a signal is acquired before the laser pulse by using two channels of the same timer in the microcontroller, as discussed earlier. Consequently, there is a possibility of dividing the samples which are selected, to subtract environment light and keep half of them before the pulse. The mathematical expression of the proposed algorithm is represented in Equation (2)—the sum of 28 points during the pulse, minus the sum of 28 points during the baseline, 14 before the pulse start and 14 after the pulse end. Also in this case, 10 points are left between the baseline and the pulse, in order to skip the transients.

(2) $$H\left(z\right)\;=\;-\overset{13}{\underset{i=0}{\sum }}z^{-i}+\;\overset{14+10+27}{\underset{i=14+10}{\sum }}z^{-i}-\overset{14+10+28+10+13}{\underset{i=14+10+28+10}{\sum }}z^{-i}\;.$$

**Figure 4:**
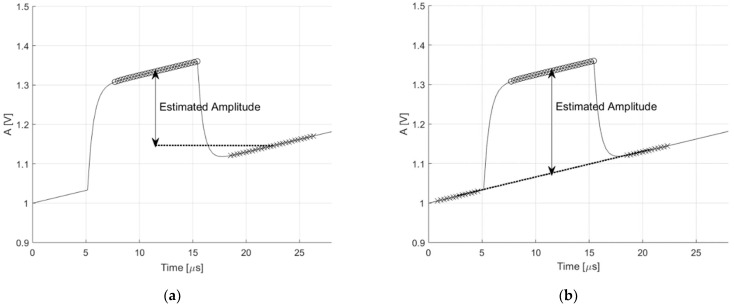
Estimated amplitude of the pulse. (**a**) Single-side digital baseline restorer and (**b**) the proposed double-side digital baseline restorer.

Figure 4 shows the comparison between the single-side digital baseline restorer (BLR) and the proposed algorithm, in the case of a ramp signal being added. As shown in Figure 4b, the enhanced performance in terms of interference immunity to compare with the second approach is achieved.

**Figure 5:**
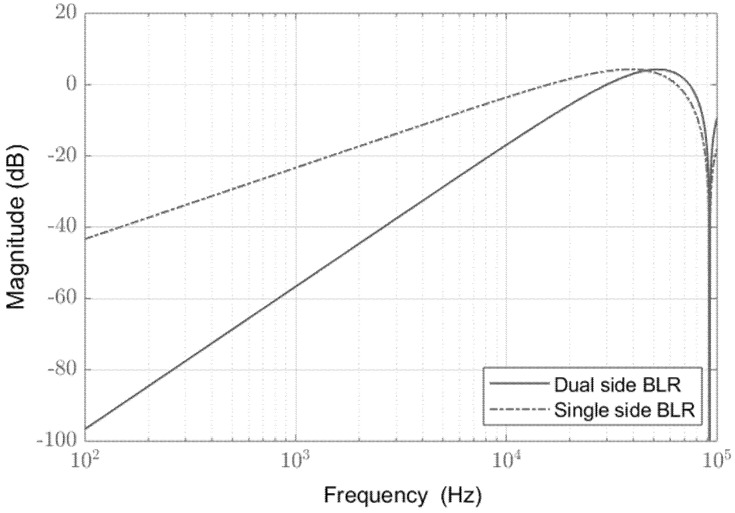
Frequency response for the single-side and the proposed double-side digital baseline restorer.

To better describe the filtering action, the frequency response of single- and double-side digital baseline restorers is represented in Figure 5 in a range up to 100 kHz. The superiority of the proposed algorithm is justified by the presence of a second zero at the origin since the slope passed from +20 dB/dec to +40 dB/dec. This agrees with the behavior of the two filters in the presence of a constant additive light and sinusoidal light interference. It is well-know that a single zero at origin in the Laplace domain means a first order derivative operation in the time domain. Therefore, it is enough to eliminate a constant light interference. In the proposed sensor, any sinusoidal interference of frequency lower than the laser pulse repetition rate can be considered as a ramp superimposed to the pulse signal, by looking only at small-time window around the received pulse. Therefore, it can be removed by the proposed algorithm by implemented the second-order derivative operation.

## 3. Measurement Results

**Figure 6:**
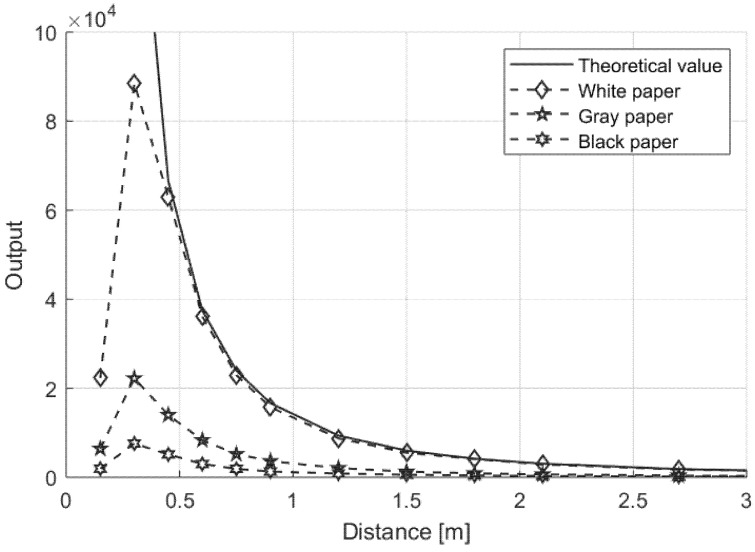
Measurement results as a function of target distance for white, gray, and black paper.

As mentioned earlier, measurement is based on evaluating the amount of reflected light from the target’s surface. Utilizing objects with different reflectivity during characterization results in developing a sensor to detect not only the object but to also extract its features. A Lambertian reflector is characterized by a surface with isotropic luminance and a diffuse light intensity related to the incident ray intensity through the cosine of the angle between the normal surface direction and the direction of the incoming light. Here, some approximations in the power budget should be considered. In the proposed sensor, the working range is much greater than the distance between the optical axis of the receiver and transmitter lenses (20 mm). This allows accounting for the transmitter and receiver lenses on the same optical path, considering that the cosine error on the whole measurement range is negligible. Further simplification is possible due to the wide range of the measurement compared to the focal length of the receiver lens and to the size of the sensitive area of the BPW34. Since the photodiode is mounted in the focal point of the receiver lens, moving the target along the measurement range leads to enlargement and lateral shift of the spotlight on its surface, as expected. However, it is always inside the sensitive area. Consequently, it can be assumed that all reflected light on lens surface is collected by photodiode. By following Equation (3), the photodiode current can be estimated.

(3) $$I_{ph}\left(r\right)\;=\;P_{T}*R_{\lambda }*\frac{d_{L}{}^{2}}{8r^{2}}$$

where $P_{t}\;\mathrm{is}\;\mathrm{the}\;\mathrm{transmitted}\;\mathrm{optical}\;\mathrm{power},\;R_{\lambda }\;\mathrm{is}\;\mathrm{the}\;$ silicon responsivity at 650 nm, $d_{L}\;\mathrm{is}\;\mathrm{the}\;$ lens diameter, *r* is the distance between the sensor and the target, the target surface reflectivity is 100%, and the speckle effect is neglected. Performance of the proposed sensor was evaluated considering the amount of light collected from a white, gray, and black paper targets, at different distances. In Figure 6, experimental results are compared to estimated values on different targets at different distances. The numerical value of the output is given by Equation (3), where every value is the output of the 12-bit converter. As results show, experimental values were in good agreement for theoretical reflection of a white paper with almost 90% of reflectivity. It should be noted that discrepancies between estimated and real values in first points were due to the optical geometry of the sensor—the reflected light was partialized because the transmitter and receiver were not on the same axis and the close target produced a focal point behind the photodiode (its position is optimized for long distances). Since the amount of the reflected light is a function of the distance, the reduction in the amplitude is expected. Considering that the noise floor of the sensor showed a standard deviation lower than 5 counts (output value measured without any target, after a simple offset cancellation), the sensor was able to correctly measure white target at a distance of up to 20 m, when the measured value was equal to about 100. No effects were noticeable from ambient light, both natural and artificial. The sensor is designed to prevent saturation even in the case of sunny conditions. The reflectivity measurement for red light also allows detection of a surface color change, if there is a corresponding variation in reflectivity for red light (for example, changing from a red to a green surface).

**Figure 7:**
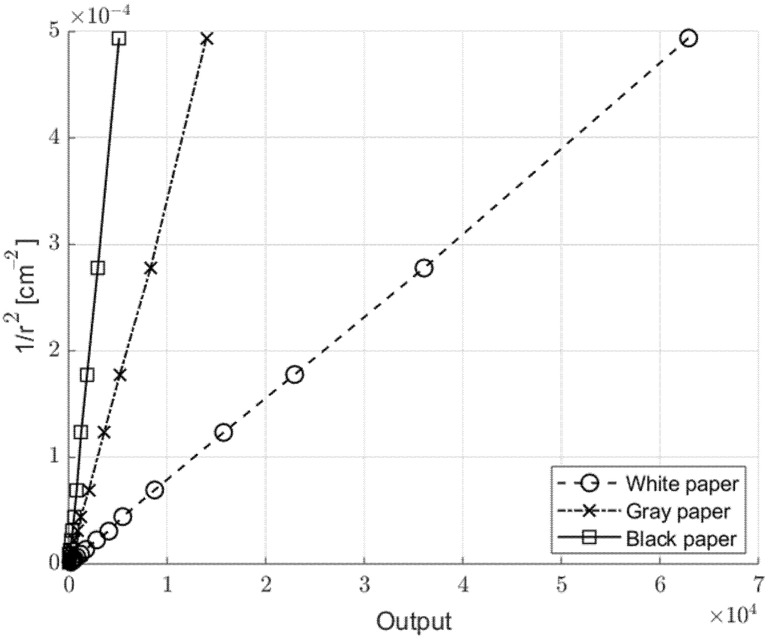
Sensor linearization curve for absolute distance measurement, for different targets—the output was proportional to the inverse of the squared distance *r*.

In addition to objects’ feature extraction, like reflectivity or color, the distance of a specific object can be estimated, if the object’s properties are known. Considering that the reflected light is inversely proportional to the squared distance, with the same target surface this monotone dependence can be linearized. Figure 7 shows the experimental dependence of the sensor output with the inverse of the squared target distance, *r*, for three different targets. The linearity is confirmed and with a simple calibration on the used target, the proposed sensor can determine the absolute distance.

For estimating the uncertainty in distance measurement, we can model the relation between numerical output, *Y*, and target distance, *r*, with a simplified equation:

(4) $$Y\left(r\right)\;=\;\frac{C}{r^{2}}\;,$$

where *C* is a proportional coefficient depending on target color, reflectivity, speckle effect and electronic gain. Therefore, the distance is given by

(5) $$r\;=\;\sqrt{\frac{C}{Y}}\;,$$

Considering this simple relation, the relative uncertainty of the distance, $u_{r}\left(r\right),$ is given by the quadratic sum of the two contribution, with coefficients due to the square root dependence, as described in [25]:

(6) $$u_{r}\left(r\right)\;=\;\sqrt{\frac{1}{4}u_{r}^{2}\left(C\right)+\frac{1}{4}u_{r}^{2}\left(Y\right)}\;\;.$$

The relative uncertainty $u_{r}\left(C\right)$ of the coefficient is almost constant and depends mainly on the target surface properties, while the absolute uncertainty on the output $u\left(Y\right)$ is almost constant, and depends mainly on disturbances and noise. The quantization uncertainty is negligible, due to the averaging of Equation (2). For these reasons, we can rewrite Equation (6) as

(7) $$u_{r}\left(r\right)\;=\;\frac{1}{2}\sqrt{u_{r}^{2}\left(C\right)+\frac{u_{}^{2}\left(Y\right)r^{4}}{C^{2}}}\;\;.$$

**Figure 8:**
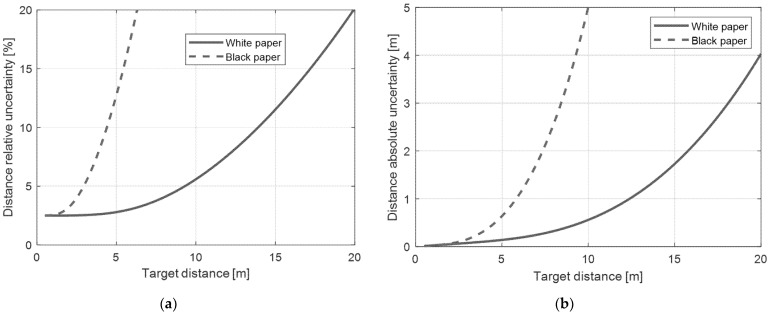
Estimated distance uncertainty as a function of target distance. (**a**) Relative uncertainty and (**b**) absolute uncertainty.

We have to underline that $u\left(Y\right)$ is strongly reduced by the digital filtering described in Equation (2), therefore for short distances, the first term in Equation (7) dominates. It indicates that the relative uncertainty in distance measurements is almost constant up to a certain distance, depending on the surface reflectivity. For example, on a white target, we experimentally measured $u_{r}\left(C\right)≅5\%$, while $u\left(Y\right)$ was measured as the output standard deviation on a fixed target, equal to about 15 counts. The value of $u\left(Y\right)$ was quite constant with distance, mainly due to electronic noise and disturbances, independent of the signal level. The optical shot-noise contribution becomes relevant in $u\left(Y\right)$ only for short distances (higher optical power received) when the contribution of $u_{r}\left(C\right)$ is dominant. Figure 8 shows the relative and absolute accuracy for white and black paper. For distances up to 4 m, the standard deviation of the target reflectivity dominates for a white target and we get about 2.5% of distance accuracy. For a black target, already at a distance of 1 m the noise on the output *Y* starts dominating, due to the low level of the signal. Obviously, if the target reflectivity is not uniform and its variability is higher than 5%, the accuracy worsens considerably. In conclusion, the application as a distance sensor does not show good performance, in terms of accuracy, resolution, or reliability, and is strongly worsened with distance. However, for some applications it could be adequate, like for proximity measurement, considering the very-low cost of the sensor. Moreover, the ability to estimate the target distance could be an addition that can be useful for particular applications, but with incomparable performance to a simple laser rangefinder. In the following, are shown the most realistic applications for this type of sensor.

**Figure 9:**
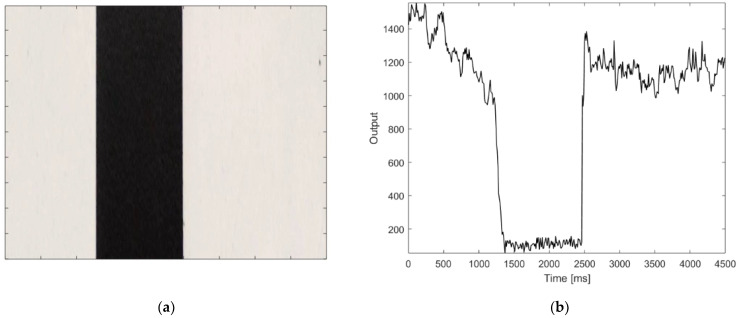
Application of the proposed sensor as remote optical encoder. (**a**) Code scale realized by a black tape and (**b**) the response of the sensor regarding the code scale measured at a distance of 6 m.

In addition to the object recognition and position detection, due to its long range, the proposed instrument can be used as remote encoder. The long distance from the object to be detected is a main concern in many industrial applications, in harsh environments, or where it is not possible to access the measurement area. Figure 9a shows a code scale, realized by a black tape attached to a white surface and Figure 9b illustrates the response of the sensor, while scanning the code scale placed at a distance of 6 m. As shown in this figure, the black target reflectivity is an order of magnitude lower that the white background, and can be located by a simple threshold algorithm, already implemented in the microcontroller.

**Figure 10:**
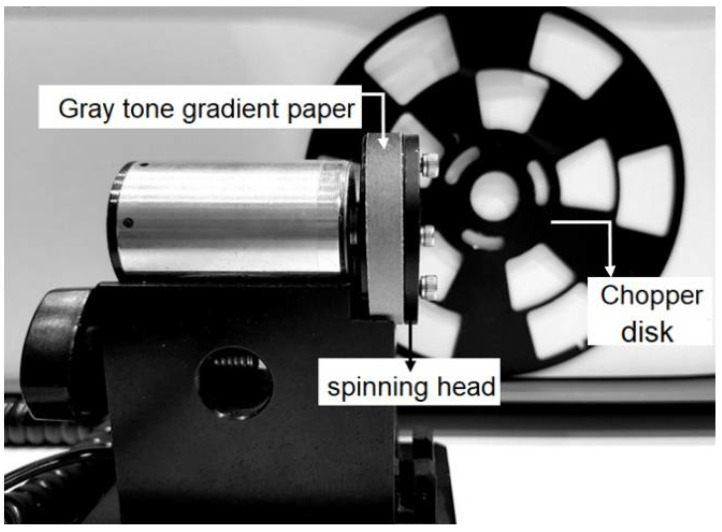
Attached gray tone gradient paper on spinning head and chopper disk used as targets for remote encoder application.

Further steps were considered to evaluate the performance of the proposed sensor as a remote encoder. To perform this, a chopper disk and spinning gray tone gradient targets were used, as shown in Figure 10. The frequency was adjusted through a chopper controller (model SR540).

**Figure 11:**
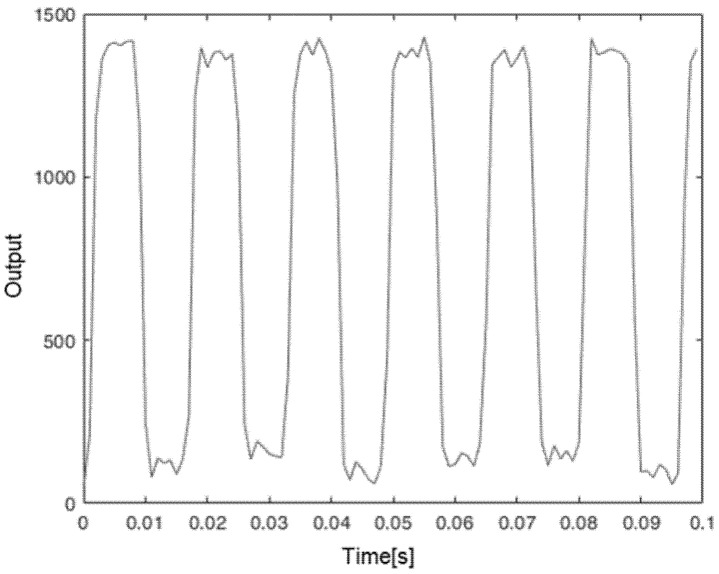
The output of the proposed sensor vs. time for the chopper disk placed 6.5 m from the sensor.

Figure 11 shows the output of the proposed sensor for the chopper disk placed 6.5 m from the sensor, with a frequency of 63 Hz. It is clear that the sensor can easily distinguish between the two states. In this case, the microcontroller was programmed with a threshold of 500, and the sensor automatically provided the digital open-collector output as a normal encoder. A similar application was tried in an industrial environment, to measure the passage of a white colored mark on a steel pipe placed about 10 m away. The steel pipe was rotating while being sprayed with water to cool it, and the colored mark worked as a keyphasor, used primarily to measure rotating speed and as a reference. In this industrial environment we did not have access to the USB data, but the sensor gave the rotation data directly to the control system, through the open-collector output. For this application, the proposed low-cost sensor provided the same result as the installed, very expensive, high-speed camera vision system.

**Figure 12:**
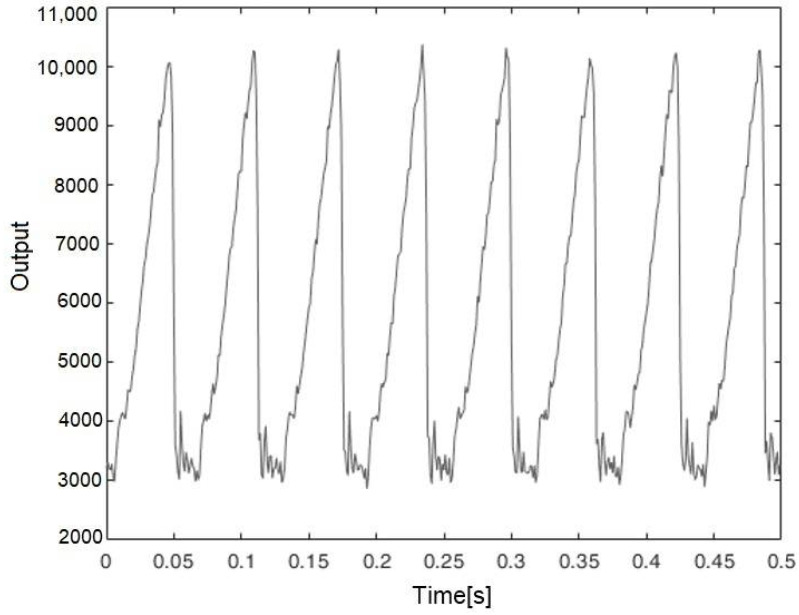
The output of the proposed sensor vs. time for the spinning gray gradient target placed 1 m from the sensor.

**Figure 13:**
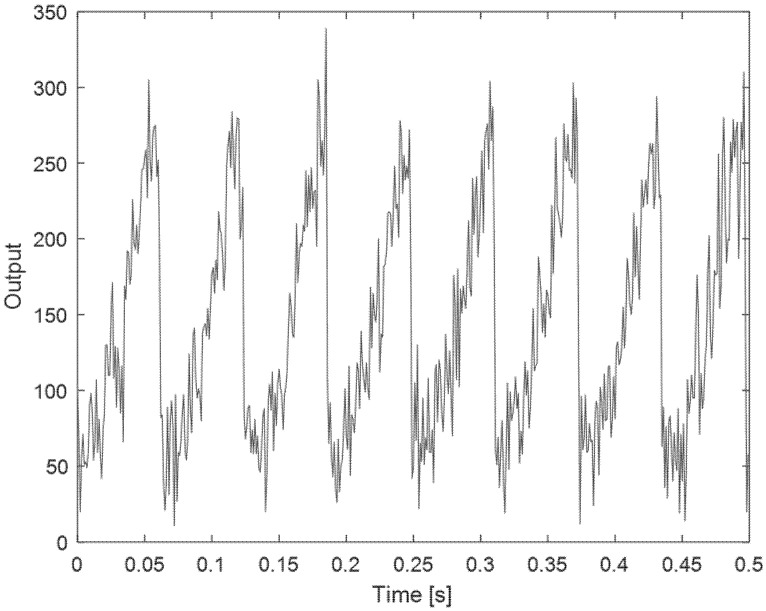
The output of the proposed sensor vs. time for the spinning gray gradient target placed 6.5 m from the sensor.

The result for the spinning gray tone gradient target, placed 1 m from the sensor, is represented in Figure 12. As shown in this figure, in addition to the high sensitivity and repeatability, a sharp transition is observed which is due to passing from pure white to pure black in gray gradient scale. From this measurement we can estimate an angle measurement with accuracy in the order of about 10°. Figure 13 shows the same measurement performed at 6.5 m of distance. As expected, the signal level was about 40 times lower, noise contribution is now evident, but the sensor still operates.

## 4. Discussion

A low-cost reflectivity sensor has been demonstrated. The principle behind the proposed sensor is the measurement of the amount of reflected light from the target surface, with a strong digital filtering in order to reduce noise and avoid disturbance and ambient light contributions. To achieve optimal performance over temperature and aging, a laser diode with a built-in automatic power control was used (model ADL-65075TA4, 650 nm red laser). The laser is driven through a high-side switch by the microcontroller which manages the laser pulses generation, acquisition of signal from the analog front-end, synchronization between optical pulses, sampling, and signal elaboration. By taking advantage of microcontroller’s computational capability and setting a pulse rate of 10 kHz with a 10% duty cycle it is possible to elaborate every single pulse in real time. In this way, the safety laser Class 2 is guaranteed in addition to the sensitivity at a distance of up to 20 m. The sensor output is updated with less than 100 μs delay from the respective laser pulse. In the receiver side, the BPW34 silicon photodiode with its large sensitive area provides good tolerance in the instrument assembly phase, and improves the sensitivity for short distances, where the optics are not well aligned. In this prototype, a biconvex lens of 1 inch and focal length 1 inch is used as receiver lens; however, it is easy to design the sensor for different working distances. The main limit for very-high distances is the laser spot dimension and therefore the spatial resolution; in this prototype the spot diameter is about 10 mm at a distance of 20 m, starting from about 3 mm at 1 m. The component cost of the whole tool is in the order of 10 €, considering the production of just one prototype. The low-cost, ease of assembly, and ease of use make the proposed sensor a perfect candidate for a number of industrial and measurement applications, such as remote encoders, where distance from the target limits the measurement systems, or for detection of reference marks on remote objects. Other possible applications are piece counting, intrusion detection, photocells or remote keyphasors. The sensor is also able to estimate the target distance, if the target surface is known, but the distance measurement performances are limited, in comparison with other optical techniques (see Table 1 and Figure 8). The distance measurement capability can be useful as an addition to a different application, for example for a keyphasor it may also be important to have an estimate of the distance of the object to be measured. From a more general point of view, the proposed sensor can replace complex and expensive vision systems, when there is no availability to the measurement area, but just a punctual measurement of reflectivity is needed.

## Figures and Tables

**Table 1:** Summary of common measurement performances of different optical technologies (orders of magnitude).

Technology	Range	Accuracy	Required Field of View
Laser interferometry (displacement)	10 m	1 μm	no
Laser interferometry (absolute distance)	10 m	100 μm	no
Laser rangefinder (continuous wave)	100 m	1 mm	no
Laser rangefinder (pulsed)	1 km	1 cm	no
Laser triangulation	1 cm	1 μm	yes
	10 cm	10 μm	yes
	1 m	1 mm	yes
Line scan camera	50 mm	0.5 μm	no
Camera triangulation	10 m	1 cm	yes
